# Beliefs regarding nicotine replacement therapy among rural residing people who smoke: a step towards promoting uptake

**DOI:** 10.1016/j.pmedr.2025.103155

**Published:** 2025-06-28

**Authors:** Dana Mowls Carroll, Andy Tan, Mackenzie Differding, Olivia A. Wackowski, Dana Rubenstein, Dorothy K. Hatsukami, Devon Noonan, F. Joseph McClernon

**Affiliations:** a Division of Environmental Health Sciences, School of Public Health, University of Minnesota, Minneapolis, MN, USA; b Annenberg School for Communication, University of Pennsylvania, Philadelphia, PA, USA; c Department of Health Behavior, Society and Policy, Rutgers School of Public Health, Institute for Nicotine & Tobacco Studies, Rutgers Biomedical Health Sciences, New Brunswick, NJ, USA; d Clinical and Translational Science Institute, Duke University School of Medicine, Durham, NC, USA; e Department of Psychiatry and Behavioral Sciences, Duke University School of Medicine, Durham, NC, USA,; f Department of Psychiatry & Behavioral Sciences, Medical School, University of Minnesota, Minneapolis, MN, USA; g Duke University School of Nursing, Durham, NC, USA

**Keywords:** Tobacco, Smoking, Rural, Disparities, Treatment, Access, Beliefs, Qualitative

## Abstract

**Objective:**

Rural areas in the United States (U.S.) have a higher smoking prevalence than urban areas. This disparity is influenced by lower odds of quitting smoking in rural versus urban areas, and lower use of evidence-based treatments, including nicotine replacement therapy (NRT). To inform strategies for promoting NRT, this qualitative study elicited and ranked NRT beliefs among rural people who smoke cigarettes.

**Methods:**

In 2023, we conducted an online, semi-qualitative, elicitation survey with US rural residing adults (ages 21+) who smoke (*n* = 52), using open-ended questions to probe about: perceived advantages/disadvantages of using NRT to quit smoking and facilitators/barriers towards using NRT. Responses were coded based on belief themes and the frequencies of these themes were tabulated.

**Results:**

Leading perceived advantages of NRT for a quit attempt included help with cravings (42 %), making quitting easier (23 %) and easing withdrawal (17 %), while perceived disadvantages were concerns about becoming addicted to another product (29 %), high cost (23 %), side effects (19 %), and being ineffective (13–17 %). Leading perceived barriers to NRT use referred to high cost (52 %), negative taste (19 %), not enough nicotine (13 %), and lack of access (11 %), while leading perceived facilitators to use were free or lower cost (54 %) and better access/availability (13 %) and flavors/taste (13 %).

**Conclusions:**

Boosting NRT use among rural communities could be achieved by (1) adopting approaches to enhance the affordability and accessibility of NRT, (2) rectifying NRT misperceptions, and (3) offering guidance on the proper use of NRT and managing side effects.

## 1 Introduction

Smoking contributes to disproportionately high rates of lives lost in rural America (Bhatia et al., 2022). Directly driving these disparities is the lower odds of quitting smoking in rural vs. urban areas (odds ratio [OR] 2010 to 2020: 0.93, 95 % Confidence interval [CI]: 0.89, 0.98]) (Parker et al., 2022). Lower quitting among rural residing people who smoke (RPWS) is driven by several factors, many of which are social determinants of health (Truth Initiative, 2018; Pesko and Robarts, 2017; Buettner-Schmidt et al., 2019). These factors include people living in rural America having lower exposure to health communications (Matthews et al., 2017; Chen et al., 2019), reduced access to quality healthcare and less frequent healthcare encounters (Minnesota Department of Health, 2022; Nuako et al., 2022), and lower cessation treatment use (e.g., nicotine replacement therapy [NRT]) (Noonan et al., 2025). Regarding the latter, prior research using the 2018–2019 United States (US) representative Population Assessment of Tobacco Use and Health Study has shown the adjusted odds of using any type of cessation treatment during a past year quit attempt was 23 % lower among RPWS versus urban people who smoke (OR: 0.77; 95 % CI: 0.60, 0.99) (Noonan et al., 2025).

Boosting NRT use in the rural population is a highly promising avenue for increasing smoking cessation. NRT, such as over-the-counter nicotine patches, gum, and lozenges, temporarily replaces nicotine from cigarettes and reduces cravings and withdrawal symptoms among people trying to quit smoking (Hartmann-Boyce et al., 2018). A 2018 Cochrane Systematic Review concluded that all types of NRT increase quitting success by as much as 50–60 % over no treatment (Hartmann-Boyce et al., 2018). A 2023 Cochrane Systematic Review further concluded that combination NRT (such as lozenge plus patch), as compared to single NRT, further enhances the ability of people to quit (Theodoulou et al., 2023).

Concerted efforts are needed to promote NRT uptake among RPWS. Towards this goal, this study elicited and ranked NRT beliefs among RPWS. We followed an elicitation survey approach similar to that used by the US Food and Drug Administration (FDA) in developing “The Real Cost” youth smoking prevention campaign (Sangalang et al., 2019). Elicitation surveys can yield beliefs about behaviors that are most salient to rural people who smoke and are particularly useful in this context due to limited literature in this population. Our elicitation survey was informed by the Integrated Model of Behavioral Prediction (Yzer, 2012), which posits that performing a behavior—in this case, using NRT during a quit attempt—depends on one's beliefs regarding the advantages and disadvantages of using NRT and what would make it easier or more difficult to use NRT. The results of this study can inform public health efforts to increase NRT uptake among RPWS.

## 2 Methods

During 2023, we enrolled rural residing people in the US who were 21+ years of age, smoked at least 100 cigarettes and, at the time of the survey, smoked ≥ five CPD on at least 25 of the past 30 days. Rural status for eligibility was based on the 2023 Rural-Urban Continuum Codes (RUCCs; range = 1 to 10), which distinguishes metropolitan counties by the population size of their metro area (RUCCs 1–3) and nonmetropolitan counties by the degree of urbanization and adjacency to a metro area (RUCCs 4–10) (U.S. Department of Agriculture, 2020). Specifically, individuals residing in a RUCC ≥4 were eligible. Recruitment was facilitated through BuildClinical, a company that advertises across digital platforms (e.g., social media). Our sample size was informed by recommendations on theoretical saturation in which additional respondents do not yield new beliefs (Sangalang et al., 2019). A prior elicitation survey among people who use tobacco relied on approximately 40–100 completers per target audience (Sangalang et al., 2019). Thus, we aimed for at least 50 completers.

We employed methods at various stages to reduce duplicate and bot responses. BuildClinical created a recruitment campaign that upon clicking required the respondent to complete a Captcha, login to their email to verify ownership of the account, and upload a photo of their identification. Respondents who attempted to submit duplicate entries with the same email and/or duplicate IP addresses were excluded at this point. Respondents were not notified why they were excluded. If the prior steps were met/completed, respondents were transferred to our REDCap survey where they completed an eligibility screener and if eligible were consented to participate (*n* = 162). Next, participants were able to advance to complete the survey and provide contact information for receipt of compensation. Those who were compensated comprised our analysis sample (*n* = 52) which required providing at least one response per prompt and contact information that matched their previously uploaded photo of their identification. The University of Minnesota IRB approved this study.

Participants were shown a description and photo of NRT. Open-ended questions captured beliefs, including advantages, disadvantages, barriers, and facilitators. Beliefs were analyzed to identify themes. See Supplementary Data for details. In brief, the process involved consolidating different phrasings that expressed the same underlying belief, assigning a common theme title to each belief, and determining the frequency of these belief themes across respondents. While all themes are described in Supplementary Table 1, the results section reports on themes elicited by >10 % of the sample, as these are less likely to reflect outliers, which may not be ideal targets for increasing uptake.

## 3 Results

The sample (*n* = 52) was drawn from 25 US states, had an average age of 42.1 years, with 51.9 % female, 90.4 % White, and 54 % with more than a high school education. Placed into context with rural America (2020 Census), our sample was over-represented by White identifying persons but similar regarding the proportion who have a high school education, General Educational Development, or less education.

Average cigarettes smoked per day were 17.1 (SD: 7.8), 84.3 % smoked their first cigarette within 30 min of waking, and average interest in quitting (response options ranged from 1 ‘not at all interested’ to 10 ‘extremely interested’) was 6.6 (SD: 2.8).

### 3.1 Advantages

**Fig. 1:**
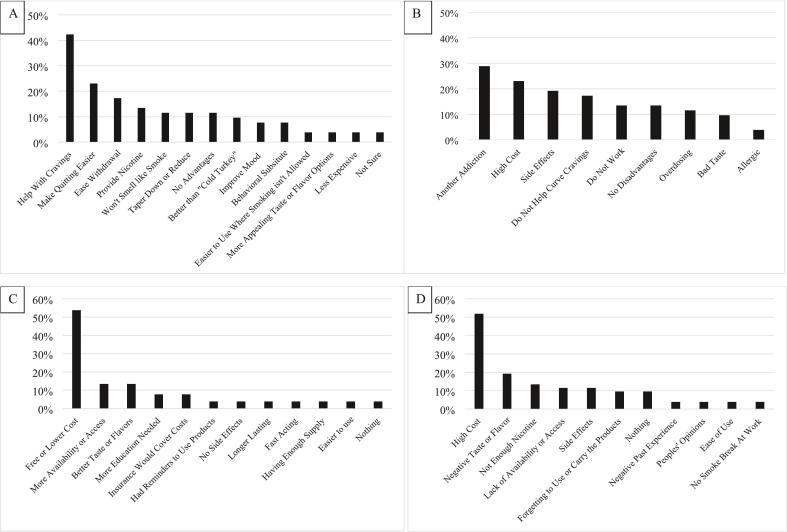
An online survey in 2023 among adults who smoke and reside in the rural United States (*n* = 52) queried about advantages (A), disadvantages (B), facilitators (C), and barriers (D) to using nicotine patches, nicotine gum, or nicotine lozenges when attempting to quit smoking. Similar responses reported by participants were classified into belief themes. The figure shows the proportion of all participants who endorsed each belief theme. Footnote: “Cold turkey” refers to quitting without any quitting medication.

The most common advantage (42 %) was that NRT could help with cravings (Fig. 1A**;** see Supplementary Fig. 1 for an alternative display of the themes). Other advantages included making quitting easier (23 %), easing withdrawal (17 %), providing nicotine (13 %), avoiding the smell of smoke (11 %), and to taper down or reduce (11 %).

### 3.2 Disadvantages

The most common disadvantage (29 %) was that NRT could lead to another addiction (Fig. 1B). For example, a participant expressed “I would just be replacing cigarettes with another form of nicotine.” Other disadvantages included the high cost (23 %), side effects (19 %), that NRT doesn't curb cravings (17 %), as well as a more general concern that NRT doesn't work (13 %), and overdosing (11 %).

### 3.3 Facilitators to use

The most common facilitator (54 %) was reducing cost (Fig. 1C), with participants suggesting that they would be more likely to use NRT if it is free or cheaper. Other facilitators included increased availability (13 %) and better taste/flavors (13 %).

### 3.4 Barriers to use

The most common barrier (52 %) was the high cost (Fig. 1D). Additional barriers included poor taste/flavors (19 %), insufficient nicotine (13 %), lack of availability (11 %) and concerns with side effects (11 %).

## 4 Discussion

In our research, RPWS reported beliefs about the advantages, disadvantages, barriers, and facilitators to using NRT. In brief, participants commonly cited that NRT could aid them in curbing cravings and making quitting easier, but had concerns about the cost, lack of availability, and the potential to continue their addiction. Additional concerns commonly noted included poor taste and side effects. This research suggests that increasing NRT among RPWS may be facilitated by (1) implementing strategies for increasing its affordability and accessibility, (2) correcting misperceptions regarding NRT, and (3) providing education on how to properly use NRT and mitigate side effects.

The research suggests a need for strategies that decrease NRT price and increase accessibility. Some health insurances cover NRT when prescribed versus purchased over-the-counter, but there are considerable limits. For example, of the 22 states who have comprehensive coverage for tobacco cessation for Medicaid enrollees, only four states as of 2024 have removed barriers to treatment access including copays, limits on treatment, and treatment duration (Centers for Disease Control and Prevention, 2024). Thus, a paradigm shift is needed in terms of approaches that decrease NRT price and increase accessibility for RPWS. Prior research tested providing a free NRT starter kit in addition to standard care in primary care settings (Dahne et al., 2020). The NRT starter kit improved 7-day abstinence in the full sample at the six month follow-up, and the effect was more pronounced in those residing in average and high rurality areas (Dahne et al., 2020). Such strategies should be further tested, particularly ones not based in primary care (i.e., direct mail, community sites) since RPWS are less likely to have routine healthcare encounters.

We also observed evidence of misperceptions about NRT among RPWS. A prior study among a general audience of people who smoke (*n* = 900) found that many participants had misperceptions about the safety and efficacy of NRT, those with misperceptions were less likely to report intent to use NRT in their next smoking quit attempt, and providing corrective information increased interest to use NRT (Ferguson et al., 2011). Recent mass media campaigns for smoking cessation have included the promotion of NRT as a component. For example, while the advertisements for the FDA's ‘Every Try Counts’ campaign do not mention NRT, the ads list a website that includes the recommendation to use NRT. Based on our findings, future research can focus on messages that specifically target rural populations with themes reinforcing the advantages of NRT while dispelling misperceptions that NRT is another form of addiction or that NRT does not work. Such research could consider the Hornik and Woolf approach (Hornik et al., 2019), which posits that beliefs are ideal targets for media campaigns when they are both strongly associated with intent to perform the behavior and are neither held by the vast majority or minority of the target audience.

We further learned that improvements in the taste of NRT would be beneficial. The NRT market has been largely unchanged in the last 10 years with orally consumed products (gum, lozenge) only available in a small range of flavors. Additional research and development is needed to develop medicinal nicotine products that are more appealing to today's consumers. To help address this gap, the FDA recently (2023) issued a final guidance for the industry titled ‘Smoking Cessation and Related Indications: Developing Nicotine Replacement Therapy Drug Products’.

There are limitations to this study. First, this was not a U.S. representative sample which means the results may be valid only for the sample tested, not the broader population of RPWS in the U.S. Additionally, rural America is not monolithic. For example, RPWS can vary in terms of prior use of NRT, which was not assessed in this study, and socioeconomic status such as educational attainment. Future research can examine beliefs across these factors as this can help identify commonalities and differences that can inform more tailored and effective strategies to boost NRT use among rural PWS. Another notable limitation is the reliance on self-report information, which means some participants could have falsely reported their smoking status or residence in a rural county.

FDA's Center for Tobacco Products' 2023 Strategic plan includes public receipt of ‘evidence-based health communication and education’. It also states to ‘educate people who use tobacco products about the benefits of cessation’. In addition, the Department of Health and Human Services published a draft framework in 2023 calling for an increase in smoking cessation efforts. The framework is built around six goals, including eliminating tobacco-related health disparities and improving the quality and accessibility of cessation services and support. Increasing NRT use among RPWS is aligned with these priorities. Research in communications and implementation science is needed to achieve these goals.

## Authorship contributions

All authors contributed to the following: Conception and design of the study, or acquisition of data, or analysis and interpretation of data; drafting the article or editing it; and approval of the submitted manuscript.

## CRediT authorship contribution statement

**Dana Mowls Carroll:** Writing – review & editing, Writing – original draft, Visualization, Supervision, Methodology, Investigation, Funding acquisition, Formal analysis, Data curation, Conceptualization. **Andy Tan:** Writing – review & editing, Writing – original draft, Methodology, Investigation. **Mackenzie Differding:** Formal analysis, Visualization, Writing – review & editing. **Olivia A. Wackowski:** Writing – review & editing. **Dana Rubenstein:** Writing – review & editing, Conceptualization. **Dorothy K. Hatsukami:** Writing – review & editing, Resources, Funding acquisition, Conceptualization. **Devon Noonan:** Writing – review & editing, Conceptualization. **F. Joseph McClernon:** Writing – review & editing, Writing – original draft, Methodology, Investigation, Conceptualization.

## Funding

Research reported in this article was supported by 10.13039/100006545NIMHD of the NIH, grants K01MD014795 and R01MD020559, and via a Masonic Cancer Center internal grant. This research was also supported by the 10.13039/100000002National Institutes of Health's 10.13039/100006108National Center for Advancing Translational Sciences, grant UL1TR002494 and TL1TR002555. The content is solely the responsibility of the authors and does not necessarily represent the official views of the National Institutes of Health.

## Declaration of competing interest

The authors declare that they have no known competing financial interests or personal relationships that could have appeared to influence the work reported in this paper.

## Appendix A Supplementary data

Supplementary material 1 Additional Details On Study Methodology. Supplementary material 1
