# Supplementary material for: Beliefs regarding nicotine replacement therapy among rural residing people who smoke: a step towards promoting uptake
Source: Prev Med Rep. 2025 Jun 28;56:103155. doi: 10.1016/j.pmedr.2025.103155 (PMC12266376; doi:10.1016/j.pmedr.2025.103155)
Supplement: Supplementary material 1 — Additional Details On Study Methodology. [file mmc1.docx]

Additional Details On Study Methodology

Prior to asking about NRT, the following description was provided along with a photo of NRT products in their original packaging: “We are now going to ask you about your beliefs about using nicotine medications such as nicotine patches, gum, or lozenges. These are approved by the U.S. Food and Drug Administration (FDA) for quitting smoking and are available over the counter (i.e., without a prescription). See the photo for examples.” Consistent with previous elicitation surveys ^1,2^, we included questions with open-ended responses because a wide range of beliefs can be generated. The exact questions are provided below. Each question had five open-ended (blank) fields for responses. The survey also collected sociodemographic (e.g., age, educational attainment) and smoking history measures (e.g., cigarettes per day, interest in quitting smoking).

Responses were analyzed jointly by a two-person team (authors DC, MD) as free-list data in Microsoft Excel, which entailed summarizing varied phrasings that relate to the same belief, generating a common belief theme title, and assessing the frequency of belief themes across respondents.^3^ We required at least two participants to express a related belief for the belief to be considered a belief theme. If a participant repeated a similar belief (e.g., wrote ‘price’ as well as ‘affordability’ in response to the same question), we counted them once for that belief theme. All free-list data (i.e., beliefs) and their associated themes were further reviewed and agreed upon by the study team (see co-authors).

Questions provided to participants:

*If you were to attempt to quit smoking, what do you see as the advantages, or good things, that might happen if you use nicotine patches, nicotine gum, or nicotine lozenges?*

*If you were to attempt to quit smoking, what do you see as the disadvantages, or bad things, that might happen if you use nicotine patches, nicotine gum, or nicotine lozenges?*

*If you were to attempt to quit smoking, what would make it easy or easier for you to use nicotine patches, nicotine gum, or nicotine lozenges if you wanted to?*

*If you were to attempt to quit smoking, what would make it difficult or more challenging for you to use nicotine patches, nicotine gum, or nicotine lozenges if you wanted to?*

Literature Cited

1. Sangalang A, Volinsky AC, Liu J, et al. Identifying Potential Campaign Themes to Prevent Youth Initiation of E-Cigarettes. *Am J Prev Med.* 2019;56(2 Suppl 1):S65-s75.

2. Brennan E, Gibson LA, Kybert-Momjian A, Liu J, Hornik RC. Promising themes for antismoking campaigns targeting youth and young adults. *Tobacco regulatory science.* 2017;3(1):29.

3. Bernard HR. *Research methods in anthropology: Qualitative and quantitative approaches.* Rowman & Littlefield; 2017.
