# Supplementary material for: Beliefs regarding nicotine replacement therapy among rural residing people who smoke: a step towards promoting uptake
Source: Prev Med Rep. 2025 Jun 28;56:103155. doi: 10.1016/j.pmedr.2025.103155 (PMC12266376; doi:10.1016/j.pmedr.2025.103155)
Supplement: Supplementary material 2 — Supplementary Figure 1. [file mmc2.docx]

| A 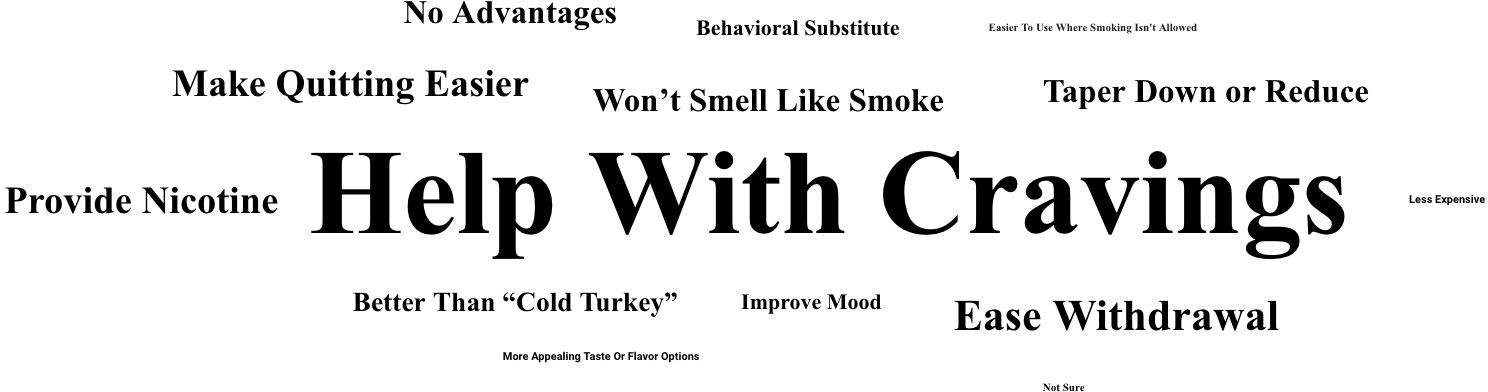 | B 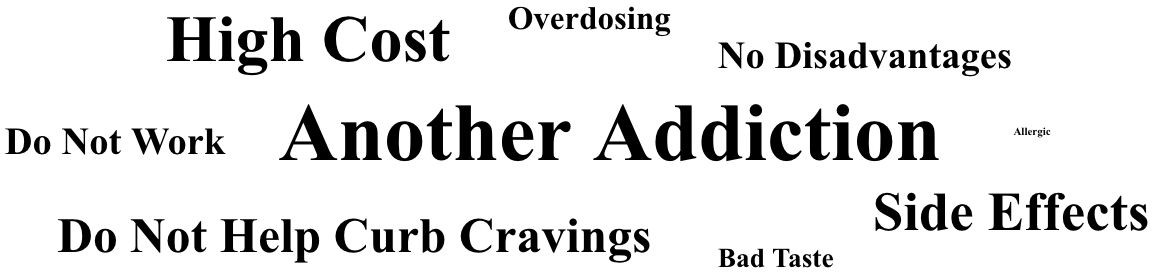 |
| --- | --- |
| C 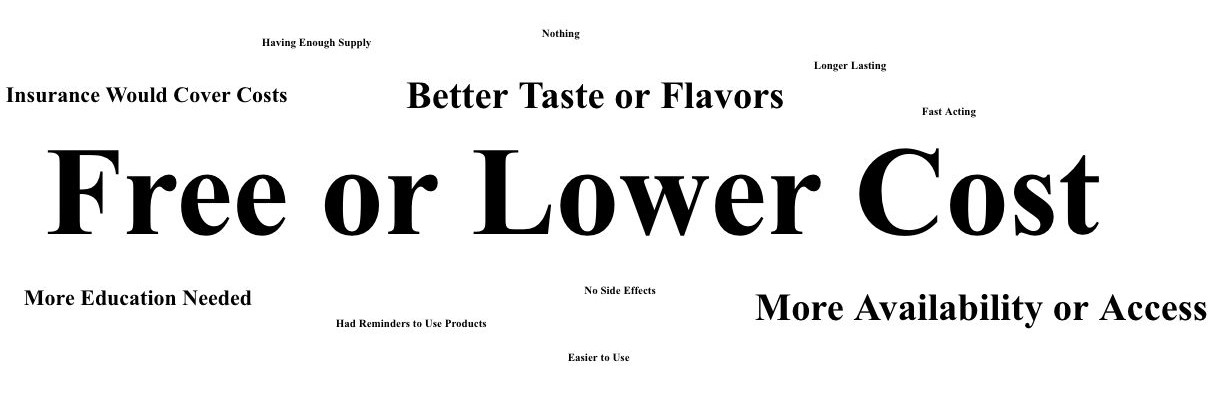 | D 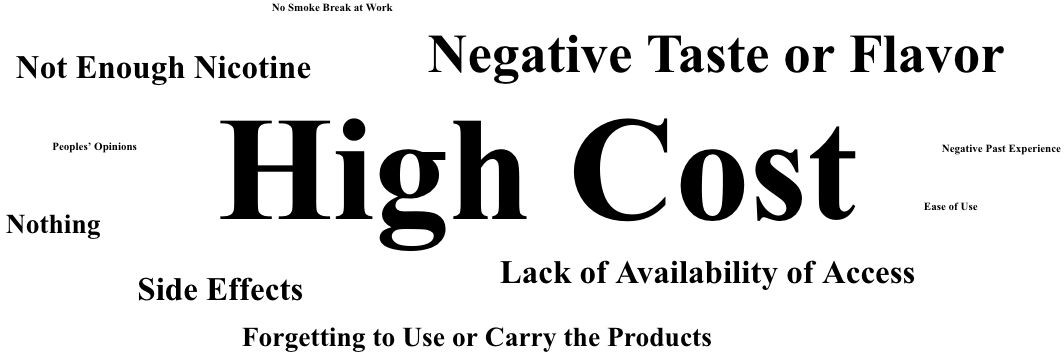 |

Supplementary Figure 1. An online survey in 2023 among adults who smoke and reside in rural United States (n=52) queried about advantages (A), disadvantages (B), facilitators (C), and barriers (D) to using nicotine patches, nicotine gum, or nicotine lozenges when attempting to quit smoking. Similar responses reported by participants were classified into belief themes. The figure shows the proportion of all participants who endorsed each belief theme.

Footnote: “Cold turkey” refers to quitting without any quitting medication.
